# The plastid genome of the critically endangered *Valeriana trinervis* (= *Centranthus trinervis*) and insights from comparison with other *Valeriana* plastomes (Caprifoliaceae)

**DOI:** 10.1007/s00425-025-04815-w

**Published:** 2025-09-10

**Authors:** Daniele De Luca, Olga De Castro

**Affiliations:** 1 Department of Humanities, University of Naples Suor Orsola Benincasa, Via Santa Caterina da Siena 37, 80132 Naples, Italy; 2 Department of Biology, University of Naples Federico II, Via Cinthia 26, 80126 Naples, Italy; 3 Botanical Garden of Naples, University of Naples Federico II, Via Foria 223, 80139 Naples, Italy

**Keywords:** Mediterranean endemic plant, Phylogenetic relationships, Plastome evolution, Whole-Genome Sequencing

## Abstract

**Main conclusion:**

The first complete plastid genome of the critically endangered species *Valeriana trinervis* was sequenced, assembled and compared with other published Valeriana plastomes.

**Abstract:**

In this study, we assembled the plastid genome of the critically endangered, endemic species *Valeriana trinervis* (= *Centranthus trinervis*) and compare it with all published plastomes of *Valeriana*. We found not only differences in the inverted repeats boundaries, in the type and abundance of repeats, but also similarities in codon usage and microsatellite numbers. We detected non-canonical start codons in several genes and identified variation in several regions that could be useful for phylogenetic and phylogeographic studies. The phylogenetic tree inference based on both full plastomes and coding sequence data indicated that *V. trinervis* is sister to all Eurasian *Valeriana* accessions confirming the phylogenetic position recently investigated. This is the first plastome available for a species of the Mediterranean clade of *Valeriana* previously known as *Centranthus*, and it adds further data to understand the evolution and diversification of this systematically debated genus.

**Supplementary Information:**

The online version contains supplementary material available at 10.1007/s00425-025-04815-w.

## Introduction

The traditional approach to plant molecular phylogenetics involves the amplification and Sanger sequencing of several variable markers (e.g. intergenic spacers, introns or genes) from different taxa (Dong et al. 2012). The plastid genome (plastome) has been always favoured over the nuclear and mitochondrial ones as source of informative molecular markers due to its small genome size (120–160 kb; Xiao-Ming et al. 2017), high copy number per cell (Morley and Nielsen 2016; Sakamoto and Takami 2018), a generally conserved structure and organization (Wicke et al. 2011), and a mutation rate suitable to phylogenies at different taxonomic levels (Gitzendanner et al. 2018). Plastid DNA sequences have been used at small scale to investigate genetic variation and gene flow within and between species (De Castro et al. 2012; Hajiahmadi et al. 2013) and for species identity (Nock et al. 2011; Dong et al. 2012; De Castro et al. 2017), and to a bigger scale to investigate relationships between genera, families, and larger groups (Li et al. 2021; Ulaszewski et al. 2021). Despite these advantages, the failure to find a universal barcode for plants or a standard set of gene markers for phylogeographic or phylogenetic studies (Cowan et al. 2006; Roy et al. 2010) has pushed the researchers towards the amplification and sequencing of larger regions (sometimes referred to as “superbarcodes”, e.g. Wu et al. 2021) or obtaining complete plastid genome sequences (Hollingsworth et al. 2016; Ji et al. 2019). Accomplices of this trend were the recent advances in sequencing technology, combined with the reduction of costs associated with high-throughput production and sequencing of DNA libraries (Tonti-Filippini et al. 2017). Although several protocols are available to enrich or amplify plastid DNA from total genomic DNA (Twyford and Ness 2017; Takamatsu et al. 2018) before carrying out high-throughput sequencing, the common procedure is to get several plastid sequences from genome-wide approaches (Dodsworth et al. 2019) or to sequence the whole plant genomes and then using bioinformatics pipelines to assemble the plastome (Freudenthal et al. 2020). In this context, a plastome can be assembled from virtually any plant genome sequencing project with adequate coverage.

In this study, we used the raw sequence data generated from a whole-genome sequencing project aimed at finding microsatellite markers for population genetic analyses in the critically endangered plant *Valeriana trinervis* Viv. (= *Centranthus trinervis* (Viv.) Bég.; Valerianoideae, Caprifoliaceae) (Di Iorio et al. 2024; De Castro et al. 2025a) to assemble and annotate the plastome of this species and compare it with those of other species of *Valeriana* L. Indeed, the phylogenetic history of Valerianoideae (Caprifoliaceae) is problematic, and several taxonomic treatments have been proposed over the years, often involving the lumping of *Centranthus* DC. in *Valeriana* (Christenhusz et al. 2018) or their recognition as distinct genera (e.g. Weberling 1961; Bell and Donoghue 2005). A recent study by De Castro et al. (2025b) aimed to test the monophyly of *Centranthus* and its relationships with *Valeriana* using a comprehensive dataset, implementing Sanger sequencing of the nuclear rRNA cistron and 10 plastid molecular markers. The results obtained identified *Centranthus* as a monophyletic group within *Valeriana* and suggested maintaining it as an infrageneric taxon under *Valeriana*. In fact, considering the position of *Centranthus*, its clade appears to be nested within a broader *Valeriana* with strong support, and given its relationship with *V. longiflora* Wilk., the separation of *Centranthus* from *Valeriana* does not seem well-supported at the present state of knowledge (De Castro et al. 2025b). Therefore, in this study we will use the accepted name of *V. trinervis* over *C. trinervis* (POWO 2025). In light of the above, the present work adds new, full plastome data to better understand the relationships and plastid features in these taxa.

## Materials and methods

### Plastome assembly and annotation

To characterise the plastome of the critically endangered *V. trinervis*, we used the Whole-Genome Sequencing (WGS) data published by us in Di Iorio et al. (2024) and available at NCBI SRA under the BioProject PRJNA1031832. Further details on sampling, DNA extraction, and library preparation are provided in the same paper. The plastome was de novo assembled using NOVOPlasty v4.3.5 (Dierckxsens et al. 2017; https://github.com/ndierckx/NOVOPlasty) by setting the configuration file with the following parameters: genome range = 120,000–180000, k-mer = 23, 6 Gb of memory, read length = 150, and insert size = 400. The plastome sequence of *V. officinalis* L. isolate B267 (GenBank accession number: NC_045052) was used as seed input. The assembly quality was evaluated as sequencing (coverage) depth (i.e. the number of sequencing reads covering each base position) following the procedure described by Ni et al. (2023), and as assembly coverage (i.e. the number of bases of all mapped reads divided by the number of bases of the reference). The circular plastid genome was first annotated with GeSeq (Tillich et al. 2017) on the CHLOROBOX platform (https://chlorobox.mpimp-golm.mpg.de/geseq.html) at default parameters. The resulting annotations were further checked by comparing them with the results of two other annotation software, PGA (Qu et al. 2019) and CPGAVAS2 (Shi et al. 2019). These three tools differ in the approach used for gene identification, specifically using BLAT-based homology searches (GeSeq), BLAST search approach (PGA), and integrating the results of multiple tools like blastn, blastx, protein2genome, and est2genome (CPGAVAS2). The results of these three tools were annotated, and then tRNA sequences were further verified using the tRNAscan-SE server v2.0 (Chan et al. 2021), and protein-coding sequences by translating the DNA sequences into amino acid sequences in MEGA X (Kumar et al. 2018) using the “Plant plastid” genetic code, followed by a blastp search (Altschul et al. 1997). In case of discordant annotations, we manually verified the start and stop codons, and checked intron and exon boundaries with the help of blast searches against closely related species. A physical map of the annotated plastome of *V. trinervis* was generated using the CPGView tool (Liu et al. 2023), which was also used as further check for annotations.

### Comparative analysis of plastid genomes

For comparative analyses of plastomes, we downloaded from NCBI the sequences (in.fasta and.gb formats) and the annotation (in.gff3 format) of the following species of *Valeriana*, for which complete plastome data were available: *V. dageletiana* Nakai ex F.Maek (PQ280047), *V. fauriei* Briq. (MW123075 and NC_065841), *V. jatamansi* Jones ex Roxb. (NC_067975), and *V. officinalis* (MW788537, NC_045052, and PQ030840). The plastid sequence NC_065841 was from Park et al. (2021), MW788537 from Li et al. (2024a, b), and NC_045052 from Wang et al. (2020a, b); all the others were not linked to any published or in press paper. Multiple accessions for the same species were kept in case of samples from different countries or when it was not possible to locate the samples with precision. The mVISTA program (Frazer et al. 2004) was used to align the above-mentioned plastid sequences in the shuffle-Lagan mode (Brudno et al. 2003) and the output was visualised with the VISTA plot tool (Mayor et al. 2000) using the plastome of *V. trinervis* from this study as reference and the default visualisation settings. To assess the degree of similarity between plastomes, we calculated the pair-wise percentage of identity using blastn (Altschul et al. 1990). Later, a second analysis in mVISTA was carried out to align the plastomes of *V. trinervis* and *Valeriana* spp. to other Valerianoideae for subsequent phylogenetic analyses: *Fedia cornucopiae* (L.) Gaertn. (= *Valeriana cornucopiae* L.) (NC_065839), *Nardostachys jatamansi* (D.Don) DC. (NC_054306), *Patrinia heterophylla* Bunge (NC_045047), and *Valerianella locusta* (L.) Laterr. (= *Valeriana locusta* L.) (NC_065842). The sequences NC_054306, NC_065839, and NC_065842 were from Park et al. (2021), while NC_045047 from Wang et al. (2020a, b).

The CPJSdraw software (Li et al. 2023) was used to visualise the junction sites of *V. trinervis* and other *Valeriana* spp. plastid genomes. Before running the software, to avoid misleading conclusions caused by non-standardised annotations, we compared the annotations of *Valeriana* plastomes downloaded from NCBI with the ones generated by CPGAVAS2, GeSeq, and PGA. To identify hotspots of variation between the above-mentioned taxa, we calculated the nucleotide diversity (Pi) in DnaSP v6.12.03 (Rozas et al. 2017) using a window length of 500 and a step size of 250 bp. In addition, Pi values were also calculated for the LSC (Large Single Copy), SSC (Small Single Copy), and IR (Inverted Repeat) regions.

The codon usage (i.e. the relative frequency of synonymous codons used to encode amino acids) for all protein-coding sequences was calculated by estimating the relative synonymous codon usage (RCSU) values for each codon. This index refers to the ratio of the actual usage frequency of a particular codon to the expected frequency when it is used without bias. A RSCU = 1 indicates no preference for that codon usage pattern, a RSCU > 1 indicates a preference (codons used more frequently than expected), while a RSCU < 1 indicates unpreferred codons. Codons with RSCU ≥ 1.5 and ≤ 0.5 are considered as high-frequency and low-frequency codons, respectively. The RSCU was calculated in the software MEGA X (Kumar et al. 2018), setting the genetic code to “Plant plastid”, for V*. trinervis* and other *Valeriana* spp.; for the latter taxa, we downloaded the coding sequences (CDSs) from GenBank using the accession numbers.

### Characterisation of plastid repeat sequences and simple sequence repeats (SSRs)

The program REPuter (Kurtz et al. 2001; https://bibiserv.cebitec.uni-bielefeld.de/reputer?id=reputer_view_submission) was used to identify and locate forward, reverse, complemented, and reverse complemented repeats in the plastid genomes of *V. trinervis* and its congenerics*.* For repeat identification, the following constraints were set: minimum repeat size of 12 bp, Hamming distance of 3 (which roughly corresponds to sequence identity ≥ 90%), and significance scores (E-values) ≥ 1e-05. Short tandem repeats (STRs) ≤ 6 bp were predicted using MISA (Beier et al. 2017) with the default parameters, i.e. repeat units ≥ 10 for mononucleotide SSRs, ≥ 6 for dinucleotide, and ≥ 5 for tri-, tetra-, penta-, and hexanucleotide SSRs; long tandem repeats (LTRs) ≥ 7 bp were predicted using Tandem Repeats Finder (TRF; Benson 1999) at the following parameters: match weight = 2, mismatch penalty = 7, delta = 7, minimum score to report a repeat (PM) = 80, minimum period size (PI) = 10, maximum period size = 50, maximum overlap between sequences = 500, and -f -d -m options activated.

### Phylogenetic inference

To assess the phylogenetic position of *V. trinervis* in respect to other species of *Valeriana* and other members of the subfamily Valerianoideae for which plastome data were available (*Fedia* Adans, *Nardostachys* DC., *Patrinia* Juss., and *Valerianella* Mill.), we carried out a Maximum Likelihood (ML) and Bayesian (BI) tree inference. First, the best evolutionary model for both full plastome data and only CDS data was selected in ModelFinder (Kalyaanamoorthy et al. 2017) implemented in IQ-Tree v2.3.6 (Minh et al. 2020) with the Bayesian Information Criterion (BIC; Stone 1979) at the settings *-m* MFP + MERGE and *-mset* mrbayes; a different partition was created for coding and non-coding regions, and for the former the partition scheme took into account the three different codon positions. Then, the ML analysis was carried out in IQ-Tree software using 1000 ultrafast bootstrap replicates (Hoang et al. 2018), while the BI tree inference was done in MrBayes v3.2.7 (Ronquist et al. 2012), running two replica runs and four chains for 1,000,000 generations and sampling chains every 1000 steps. Convergence and effective sample sizes (ESS) for all parameters of BI analysis were checked in Tracer v1.7 (Rambaut et al. 2018), and the latter was considered acceptable when > 200. In addition, the first 10% of samples was discarded as burn-in. Phylogenetic trees were visualised using FigTree v1.4.3 (http://tree.bio.ed.ac.uk/software/figtree). The plastome of *P. heterophylla* was chosen as outgroup.

## Results

### Assembly of the plastome of *V. trinervis* and its characteristics

**Fig. 1:**
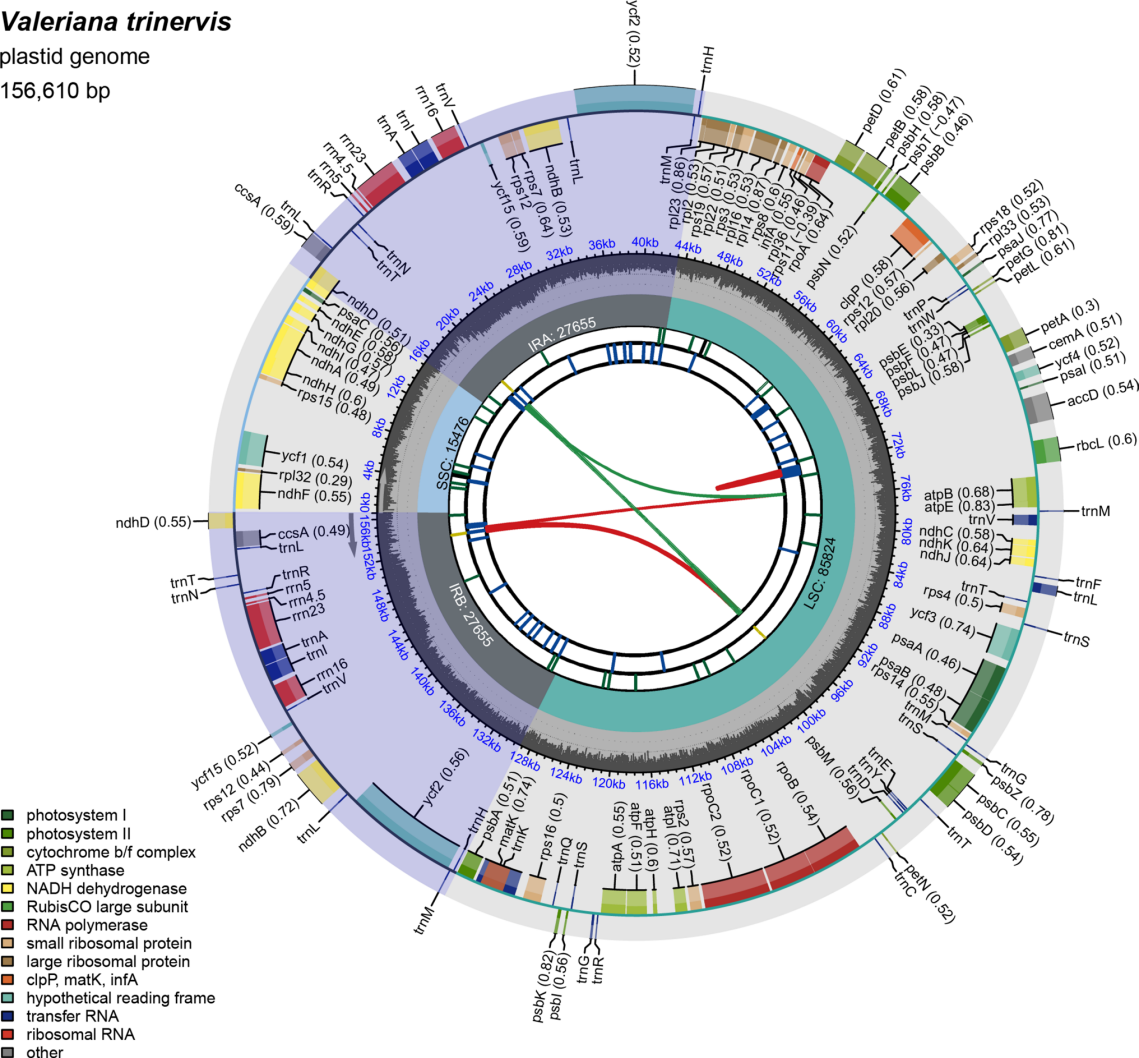
Map of the plastid genome of *Valeriana trinervis* (Caprifoliaceae). In the six tracks from the centre outward are indicated: (i) dispersed repeats: in green palindromic and in red direct repeats; (ii) long tandem repeats (short blue bars); (iii) short tandem repeats or microsatellite sequences (short bars in black = complex repeats; green = mono-repeats; yellow = di-repeats); (iv) small single-copy (SSC), inverted repeat (IRA and IRB), and large single-copy (LSC) regions; (v) GC content along the genome; (vi) genes

The total number of reads utilised for the assembly of the plastome was 9,998,176, of which 354,454 were successfully aligned to the assembly graph, and 240,782 were incorporated into the final assembly with an average coverage of 469 × . The plot of the sequencing depth (Supp. Information S1 – Suppl. Fig. S1) indicated that the aligned reads had a minimum sequencing depth of 353 × for the full-length plastome; the average sequencing depth (7760 ×) was not markedly different from the maximum sequencing depth (7998 ×), indicating no differences in the coverage of bases with the increase of contig size. The plastome of *V. trinervis* was circularised into a sequence of 156,610 bp, showing the typical quadripartite structure composed by a large single copy region (LSC), a small single-copy region (SSC) and a pair of inverted sequences in the opposite orientation (IRa and IRb) separating the two single-copy regions (Fig. 1). The LSC region was 85,824 bp long, the SSC was 15,476 bp long, and the inverted repeat region (IRa + IRb) was 55,310 bp long. The plastome sequence is available in GenBank under accession number PV026107.

A total of 136 gene regions were annotated, corresponding to 113 unique (not duplicated) genes distributed as follows: 76 protein-coding genes, 4 rRNAs, 28 tRNAs, and 5 genes of unknown function (Table 1). Among the duplicated genes were *ccs*A, *ndh*B, *ndh*D, all the rRNAs (*rrn*4.5, *rrn*5, *rrn*16, and *rrn*23), *rps*7, *rps*12, the tRNAs *trn*A-UGC, *trn*H-GUG, *trn*I-GAU, *trn*L-CAA, *trn*L-UAG, *trn*N-GUU, *trn*R-ACG, *trn*V-GAC, and the genes with unknown function/ open reading frames *ycf*2 and *ycf*15; the tRNA-coding genes *trn*M-CAU and *trn*T-GGU were found in three copies. Introns were annotated in 17 genes: 15 of them contained one intron, while two (*clp*P and *ycf*3) two introns. The *rps*12 gene, encoding the ribosomal small subunit 12, was trans-spliced into three exons, of which one separated by around 28 kb from the other two and with a complement region far around 81 kb; two copies were annotated in the plastome, one of 345 bp and another one of 369 bp. The shorter copy (8 aa less) ended with the unusual triplet AGT. We exclude mis-annotations because the following 24 bp or 8 aa had no similarity with the full sequences of other *Valeriana* spp. or the other copy in *V. trinervis*. The *ndh*D gene was duplicated and presented a non-canonical starting codon in both copies: ACG instead of AUG. In addition, the two copies were different in size (912 bp and 1509 bp).

**Table 1:** List of the genes annotated in the plastome of *Valeriana trinervis*. *Gene with one intron; **Gene with two introns; (× n) indicates the number of copies

Functional category	Localization/ Function	Name	Total
Photosynthesis	ATP synthase	*atp*A*, atp*B, *atp*E, *atp*F*, *atp*H, *atp*I	6
	Cytochrome b/f complex	*pet*A, *pet*B*, *pet*D*, *pet*G, *pet*L, *pet*N	6
	NADH dehydrogenase	*ndh*A*, *ndh*B* (× 2), *ndh*C, *ndh*D (× 2), *ndh*E, *ndh*F, *ndh*G, *ndh*H, *ndh*I, *ndh*J, *ndh*K	11
	Photosystem I	*psa*A, *psa*B, *psa*C, *psa*I, *psa*J	5
	Photosystem II	*psb*A, *psb*B, *psb*C, *psb*D, *psb*E, *psb*F, *psb*G, *psb*H, *psb*I, *psb*J, *psb*K, *psb*L, *psb*M, *psb*N, *psb*T, *psb*Z	16
	Rubisco large subunit	*rbc*L	1
Self-replication	DNA-dependent RNA polymerase	*rpo*A, *rpo*B, *rpo*C1*, *rpo*C2	4
	Ribosomal RNAs	*rrn*4.5 (× 2), *rrn*5 (× 2), *rrn*16 (× 2), *rrn*23 (× 2)	4
	Ribosome large subunits	*rpl*2*, *rpl*14, *rpl*16*, *rpl*20, *rpl*22, *rpl*23, *rpl*32, *rpl*33, *rpl*36	9
	Ribosome small subunits	*rps*2, *rps*3, *rps*4, *rps*7 (× 2), *rps*8, *rps*11, *rps*12* (× 2), *rps*14, *rps*15, *rps*16*, *rps*18, *rps*19	12
	Transfer RNAs	*trn*A-UGC*(× 2), *trn*C-GCA, *trn*D-GUC, *trn*E-UUC, *trn*F-GAA, *trn*G-GCC, *trn*G-UCC*, *trn*H-GUG (× 2), *trn*I-GAU*(× 2), *trn*K-UUU, *trn*L-CAA (× 2), *trn*L-UAA*, *trn*L-UAG* (× 2), *trn*M-CAU (× 3), *trn*N-GUU (× 2), *trn*P-UGG, *trn*Q-UUG, *trn*R-ACG (× 2), *trn*R-UCU, *trn*S-GCU, *trn*S-GGA, *trn*S-UGA, *trn*T-GGU (× 3), *trn*T-UGU, *trn*V-GAC (× 2), *trn*V-UAC*, *trn*W-CCA, *trn*Y-GUA	28
Other genes	Acetyl-CoA carboxylase	*acc*D	1
	C-type cytochrome synthesis gene	*ccs*A (× 2)	1
	Envelope membrane protein	*cem*A	1
	Maturase	*mat*K	1
	Protease	*clp*P**	1
	Translation initiation factor	*inf*A	1
	Hypothetical open reading frames	*ycf*1, *ycf*2 (× 2), *ycf*3**, *ycf*4, *ycf*15 (× 2)	5

The investigation of codon usage preferences showed a high preference (RSCU ≥ 1.5) for the following 12 codons: AAU(N), ACU(T), AGA(R), CAU(H), GAU(D), GCU(A), GGA(G), GUU(V), UAA(*), UAU(Y), UCU(S), and UUA(L). Instead, 11 codons were less abundant, indicating negative codon usage bias (RSCU and ≤ 0.5): AAC(N), ACG(T), AGC(S), CGC(R), CGG(R), CUC(L), CUG(L), GAC(D), GCG(A), GGC(G), and UAC(Y). Two codons, AUG(M) and UGG(W), were considered neutral, being used approximately equally to all synonymous codons (Supp. Information S2–Suppl. Table S1).

The repeat elements detected beyond the chosen threshold were 646, of which 558 (86.4%) as forward repeats, 86 (13.3%) as palindromic repeats, and 2 (0.3%) as reverse repeats. Regarding microsatellite regions, we found 39 SSRs, of which five in compound formation, mostly localised in the LSC (19) and SSC (10) regions. Only five repeats (four mono- and one dinucleotides) were observed in each IR region (Supp. Information S2–Suppl. Table S2). The mono-repeat A/T was the most abundant repetition, accounting for the 87.2% (34/39) of all microsatellites (Supp. Information S2–Suppl. Table S3), followed by the dinucleotide repeats AT/AT (7.7%; 3/39), and the mononucleotide repeats G/C and AC/GT (both 2.6%; 1/39). Most of cpSSRs were located in intergenic regions, except for six that were found within genes (*rpo*B, *rpo*C1, *ycf*1, and *ycf*2) and other six within introns (in *atp*F, in the second intron of *clp*P, in *rpl*16, *rpo*C1, *trn*G-UCC, and *trn*I-GAU).

### Comparative genomics analysis with other *Valeriana* spp.

The plastome of *V. trinervis* was 569 bp longer than the longest plastome of *V. officinalis* (PQ030840) and ~ 6 kb longer than the shortest plastome (*V. officinalis* voucher SN0759, MW788537) (Table 2). Such length differences mostly occurred in conserved non-coding sequences (CNSs) (Supp. Information S1 – Suppl. Fig. S2). The total GC content of the analysed plastomes was comparable across the taxa investigated, setting at 38%. Some differences for GC% were observed in the SSC and IR regions, with *V. officinalis* vouchers B267 and SN0759 showing a higher GC content for IRs and SSC and IRs, respectively (Table 2). In terms of overall similarity, the plastome of *V. trinervis* was identical at the 96–97% to the other *Valeriana* plastomes (Supp. Information S2 – Suppl. Table S4).

**Table 2:** Characteristics of the plastome of *Valeriana trinervis* and comparisons with other *Valeriana* spp. plastomes. N.a. stands for “not available”; *, according to POWO (2025)

	*V. trinervis*	*V.* *dageletiana*	*V. fauriei*	*V. fauriei* PDBK	*V. jatamansi*	*V. officinalis*	*V. officinalis* B267	*V. officinalis* SN0759
GenBank accession number	PV026107 (this study)	PQ280047	NC_065841	MW123075	NC_067975	PQ030840	NC_045052	MW788537
Geographic location	Corsica (France)	South Korea	n.a.	n.a.	China	Kazakhstan	China	China
Native geographic distribution*	Corsica (France)	Europe	Russian Far East to temperate East Asia	Russian Far East to temperate East Asia	Himalaya to W. & Central China and N. Myanmar	Europe to NW. Iran	Europe to NW. Iran	Europe to NW. Iran
Total length (bp)	156,610	155,311	155,302	154,432	155,138	156,041	151,505	150,612
%GC	38.15	38.34	38.35	38.36	38.34	38.26	30.45	38.49
SSC length (bp)	15,476	15,195	15,159	15,199	15,108	15,250	16,288	18,339
SSC %GC	32.67	32.6	32.59	32.65	32.65	32.56	32.53	33.1
LSC length (bp)	85,824	85,478	85,541	85,295	86,368	85,191	87,619	85,547
LSC %GC	36.55	36.71	36.73	36.72	36.74	36.66	36.54	36.76
IRs length (bp)	55,310	54,728	54,602	53,938	53,662	55,600	47,598	46,726
IR %GC	42.2	42.47	42.5	42.56	42.5	42.25	43.97	43.77

The mVISTA alignment of *V. trinervis* and the other *Valeriana* plastomes (Supp. Information S1–Suppl. Fig. S2, and Supp. Information S3–Suppl. Dataset S1) showed an overall conserved structure, with most of the variation present not only in non-coding regions (e.g. *acc*D-*rbc*L, *clp*P introns, *ndh*I-*ndh*G, *psb*J-*pet*A, *trn*T-GGU- *trn*L-UAG, *trn*V-UAC-*ndh*C, *ycf*1-*rps*15) but also in coding ones (e.g. *acc*D, *pet*D, *rpo*C1, *rps*18, and *ycf*1).

Our check of *Valeriana* annotations following the procedure described in Materials and Methods enabled to properly identify non-canonical start codons for the *ndh*D gene and to annotate this gene in the plastome of *V. jatamansi*. Annotations in.gb format from CPGAVAS2 are available in Supp. Information S3 – Suppl. Datasets S2–S8. All *Valeriana* plastomes from literature contained two copies of the *ndh*D gene, except those of *V. officinalis* vouchers B267 and SN0759, which presented just one copy. As for *V. trinervis*, the two copies of *ndh*D of other *Valeriana* spp. had different length (Supp. Information S1 – Suppl. Fig. S3). The map of IR boundaries (Supp. Information S1 – Suppl. Fig. S3) showed a rather conserved gene order at the inverted repeats boundaries of *V. trinervis* and most of *Valeriana* spp. here considered; however, some exceptions also occurred. For instance, the LSC/IRb boundary (JLB) was found between *psb*A and *trn*H genes in all taxa except *V. officinalis* B267, in which *trn*M replaced *trn*H. In *V. officinalis* accession B267, *psb*A was 482 bp distant from the beginning of IRb against the 61–76 bp distance observed in the other taxa. On the other side of the boundary and in the same accession, *trn*M was 218 bp distant from the beginning of IRb, while in all the other taxa *trn*H was 122–159 bp distant (Supp. Information S1 – Suppl. Fig. S3). The junction SB (IRb-SSC) was characterised by *ndh*D and *ndh*F genes in all taxa except for two accessions of *V. officinalis*, B267 and SN0759, where *trn*N was found instead of *ndh*D. The latter gene was found within the JSB in *V. dageletiana*, the two accessions of *V. fauriei* and in one accession (PQ030840) of *V. officinalis*; in *V. trinervis, ndh*D was located at 79 bp from the JSB, while in *V. jatamansi* it was exactly at the boundary. The highest distance between a gene and the end of IRb was observed in *V. officinalis* B267 and SN0759 (269 bp and 432 bp, respectively). In the junction SSC-IRa (JSA) the genes *psa*C and *ndh*D were present in all taxa except for the two above-mentioned accessions of *V. officinalis*, which had *trn*N instead of *ndh*D. At this junction, *ndh*D was within the boundary in all the taxa in which it was present except for *V. fauriei* PDBK, where it was located at 150 bp from the junction, within the IRa. The gene *psa*C was at only 337 bp from the beginning of IRa, against the 528–654 bp distance observed in the other taxa. Finally, the IRa-LSC boundary (JLA) was between *trn*H and *rpl*23 in *V. trinervis* and all *Valeriana* accessions excluding *V. officinalis* B267, where *trn*M replaced *trn*H, and it was located at the highest distance (218 bp against 122–159 bp) from the beginning of LSC in respect to other taxa (Supp. Information S1 – Suppl. Fig. S3). Regarding expansions and contractions of IRs, no relevant differences were generally observed between *V. trinervis* (55,154 bp) and most of *Valeriana* taxa (53,662–55,600) except for two accessions of *V. officinalis*, B267 and SN0759, which showed a marked reduction in size, with 47,598 bp and 46,726 bp, respectively (Table 2, and Supp. Information S1 – Suppl. Fig. S3).

**Fig. 2:**
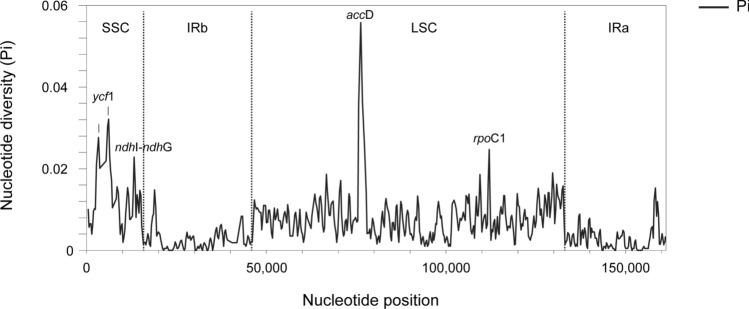
Nucleotide diversity of *Valeriana trinervis* and other *Valeriana* plastomes

The graph of nucleotide diversity (Pi) analysis carried out in DnaSP software (Fig. 2) indicated that the regions with the highest mean Pi values were the SSC (Pi = 0.012) and LSC (0.008), respectively; the IR region had a nucleotide diversity of 0.002. Within these regions, the genes/non-coding sequences with the highest Pi values were *acc*D, *ndh*I-*ndh*G, *rpo*C1, and *ycf*1.

The codon usage in *V. trinervis* was comparable to other *Valeriana* species (Supp. Information S1 – Suppl. Fig. S4). The codons GCU, UCU, and UUA, respectively corresponding to the amino acids alanine, serine, and leucine, were the ones with the highest codon bias (approaching RSCU = 2). On the contrary, the codons ACG (serine), CAC (histidine), CGC (arginine), CUG and CUC (leucine), and UAC (tyrosine) showed negative bias (were less used). The preferred stop codon was UAA (RSCU ~ 1.5), while the canonical start codon AUG (methionine) showed no usage bias (Supp. Information S1 – Suppl. Fig. S4). Overall, approximately the 47% of codons showed preferences in usage (RSCU > 1).

Non-canonical start codons were observed in the *ndh*D, *psb*C, *psb*L, and *rps*19 genes. Specifically, the *ndh*D gene presented the codon ACG (threonine) instead of ATG (methionine) as start codon in all the taxa analysed except for *V. officinalis* voucher SN0759, which presented GTG as start codon; this gene was not annotated in the plastome of *V. jatamansi*. The *psb*C gene showed the non-canonical start codon GTG only in *V. officinalis* accession PQ030840. In all the other taxa it started with ATG (methionine). The start codon for *psb*L was ACG instead of ATG in all the taxa except *V. officinalis* voucher SN0759 (TTG); it was not annotated in *V. jatamansi*. Finally, the *rps*19 gene presented the canonical start codon ATG only in *V. trinervis* and *V. jatamansi*, but was replaced by GTG in all the other taxa.

**Fig. 3:**
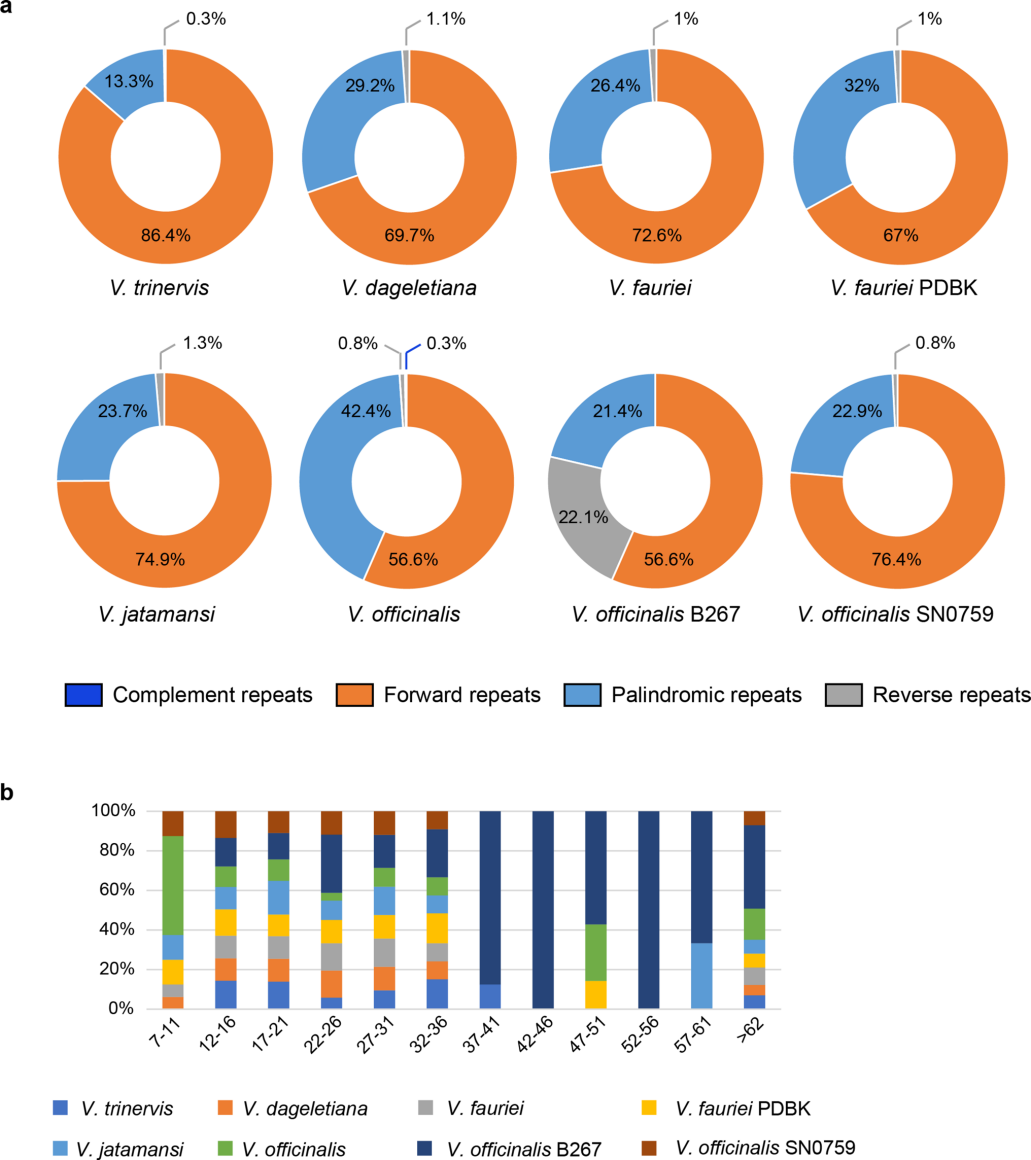
Repeat elements in the plastomes of *Valeriana trinervis* and other *Valeriana* species. **a** Complement, forward, palindromic, and reverse repeats. **b** Long tandem repeats (> 7 bp)

Regarding repeated regions, the plastome of *V. trinervis* contained the highest proportion of forward repeats (86.4%) and the lowest of palindromic repeats (13.3%) (Fig. 3a). Within the other *Valeriana* species there was high heterogeneity in the proportion of different classes of repeat elements, which was generally not consistent with taxonomy. This was particularly evident in the *V. officinalis* group, where reverse repeats were 0.3% and 0.8% in and *V. officinalis*, respectively, and 22.1% in, while palindromic repeats were 21.4% and 22.9% in *V. officinalis* voucher B267 and voucher SN0759, and 42.4% in *V. officinalis* (Fig. 3a). The sizes of long tandem repeats (LTRs) common to all taxa ranged from 12 to 36 bp (Fig. 3b, Supp. Information S2 – Suppl. Tables S5–12). *Valeriana officinalis* B267 lacked LTRs < 12 bp, but was the only taxon having them in all the size ranges considered, including exclusive sizes (e.g. for repeat classes of 42–46 bp and 52–56 bp). LTRs in the sizes between 37 and 61 bp were present in a few taxa like *V. trinervis* (37–41), *V. fauriei* and *V. officinalis* (47–51 bp), *V. jatamansi* (57–61 bp), and the above-mentioned *V. officinalis* B267. Long tandem repeats > 62 bp were present in all taxa but in scattered and mostly non-overlapping size classes (Supp. Information S2 – Suppl. Tables S5–12).

The number of microsatellite repeats (SSRs) in *V. trinervis* (39) was identical to *V. fauriei* voucher PDBK (39), and not very different from *V. officinalis* (38) and *V. dageletiana*, *V. fauriei*, and *V. officinalis* SN0759 (38); the lowest number of SSRs was observed in *V. officinalis* B267 (30) (Table 3). The motif A/T was the most recurrent in all the plastid genomes here investigated, with a length in the range of 10–17 repeats in all the taxa except *V. officinalis*, where the longest repeat was of 19 bp. *Valeriana trinervis* was the only species showing the AC/GT dinucleotide motif, while the tri-nucleotide motif AAT/ATT was only found in *V. officinalis* B267 and presented the longest repetition range (24–31). The G/C mono-repeat was found only in four taxa and was shortest in *V. trinervis* (10 bp against 12–14 in the other taxa). The AT/AT dinucleotide repeat, common to all taxa, showed the longest repetition range in *V. trinervis* (6–10 against the common 6–7 range of other *Valeriana* species).

**Table 3:** Number of cpSSRs in the plastome of *Valeriana trinervis* and of the others *Valeriana* spp. The estimates consider sequence complementarity. In parentheses is reported the minimum and maximum repeat length

	Tot SSRs	A/T	G/C	AC/GT	AT/AT	AAT/ATT
*V. trinervis*	39	34 (10–17)	1 (10)	1 (8)	3 (6–10)	0
*V. dageletiana*	37	32 (10–17)	2 (12–14)	0	3 (6–7)	0
*V. fauriei*	37	32 (10–17)	2 (12–14)	0	3 (6–7)	0
*V. fauriei* PDBK	39	36 (10–17)	0	0	3 (6–7)	0
*V. jatamansi*	36	34 (10–17)	1 (12)	0	1 (8)	0
*V. officinalis*	38	36 (10–19)	0	0	2 (6–7)	0
*V. officinalis* B267	30	25 (10–17)	0	0	3 (6–7)	2 (24–31)
*V. officinalis* SN0759	37	34 (10–17)	0	0	3 (6–7)	0

**Fig. 4:**
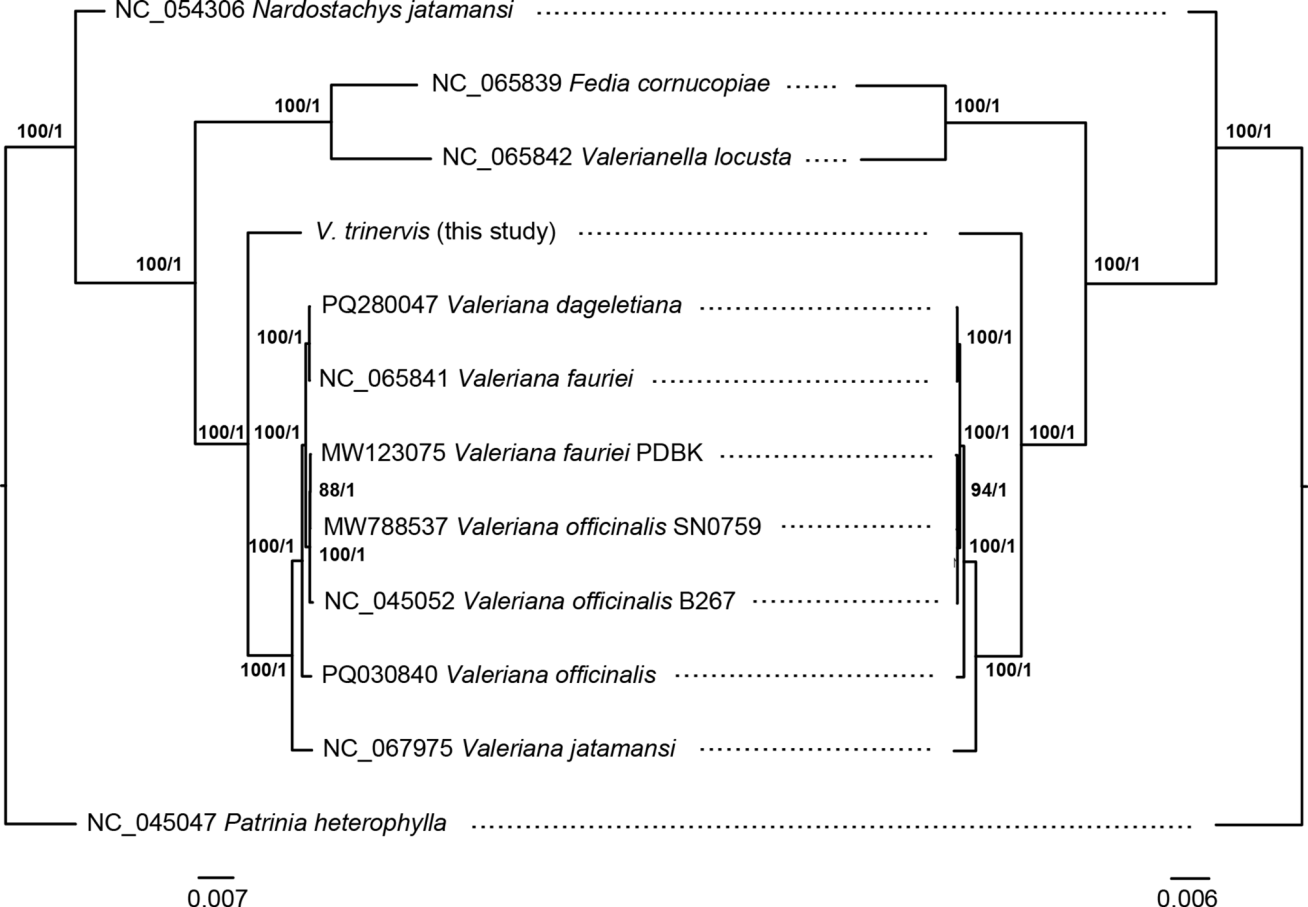
Maximum likelihood and Bayesian phylogenetic trees based on the full plastome (left) and coding sequences data (right). Numbers at nodes refer indicate support to that relationship in terms of bootstrap (before /) and posterior probability (after /)

The topology of Maximum likelihood and Bayesian phylogenetic trees was identical, as well as the topology of these trees when considering the full plastome sequences (tree on the left in Fig. 4) and the CDS-based trees (tree on the right in the same figure). All nodes were supported with a 100% bootstrap value or 1 posterior probability, except for the node connecting *V. fauriei* voucher PDBK (MW123075) and *V. officinalis* voucher SN0759 (MW788537), which had a bootstrap support of 88 in the plastome-based ML tree and 94 in the CDS-based one. Whatever the methodology and dataset used, *V. trinervis* was sister to all the other *Valeriana* spp. with maximum support (Fig. 4). The samples of *V. officinalis* and *V. fauriei* turned out to be not monophyletic.

## Discussion

In this study, we assembled, annotated, and deposited the plastid genome of the critically endangered species *V. trinervis*, a taxon previously included in the problematic *Centranthus* s.l. group (De Castro et al. 2025b). This species is found exclusively in a single location in Corsica, and has an estimated population size of less than 150 individuals (Revaka et al. 2012–2017). The plastome was fully assembled from raw sequence data originally produced to obtain a genome-wide set of nuclear microsatellite markers for population genetics studies in this species and other congenerics (Di Iorio et al. 2024; De Castro et al. 2025a). By subsampling the 4.56% of reads present in the raw data, NOVOPlasty was capable of circularising a plastome with an assembly coverage of 469 × using 6 Gb of RAM; the sequencing depth was overall high (average value of 7760 ×). In line with available literature on plastome assembly strategies (reviewed in Twyford and Ness 2017) we confirmed that these “untargeted” whole-genome sequencing (WGS) approaches can be suitable for plastome assembly, providing that data have adequate depth and that did not undergo size selection with specific restriction enzymes (e.g. in GBS or RAD approaches).

*Centranthus* is a problematic genus into an even more problematic family, the Valerianaceae, whose taxonomic validity is currently debated in favour of Caprifoliaceae (APG 2016; POWO 2025). The most recent accepted treatment (Christenhusz et al. 2018) sees the inclusion of some Valerianoideae (*Centranthus*, *Fedia*, *Valeriana*, and *Valerianella*) except *Patrinia* and *Nardostachys* into a large *Valeriana “*super-genus” (De Castro et al. 2025b). As outlined in De Castro et al. (2025b), this choice is premature on account of the current phylogenetic evidence, which would benefit from a wider taxon sampling, and considering the distinct morphological traits of some members. All the phylogenetic studies produced so far (e.g. Hidalgo et al. 2004, 2010; Bell et al. 2012, 2015) agree in retrieving *Patrinia* and, in turn, *Nardostachys*, as sister to all the other Valerianoideae; in turn, *Fedia* and *Valerianella* are sister to *Centranthus* and *Valeriana*. On the contrary, recent (De Castro et al. 2025b) and past (Hidalgo et al. 2004, 2010; Bell et al. 2015) phylogenetic studies support *Centranthus* as synonym of *Valeriana*. As consequence, we considered *C. trinervis* as any other *Valeriana*, under the name *V. trinervis*.

The full plastome and CDS-based Bayesian and Maximum likelihood trees here inferred confirm this topology with high support (Fig. 4) and retrieve *V. trinervis* as sister to the Eurasian *Valeriana* species for which plastome sequences were available. Regarding the relationships among the other *Valeriana* species, for the purposes of this study we highlight that there is no monophyly for *V. fauriei* and *V. officinalis* samples; these differences also reflect in other characteristics other than sequence differences (e.g. repeat type, see below) and could be due to real differences in samples or erroneous taxonomic determination. Indeed, other studies (Fujii et al. 2021; Bączek et al. 2022) have found intraspecific diversity in these taxa, that could be explained with the wide geographic distribution. From a phylogenetic perspective, we cannot argue more about the relationships among *Valeriana* plastomes because most of taxa retrieved from literature and included in this study (*V. fauriei*, *V. jatamansi*, and *V. officinalis*) are all in the so called “Eurasian group” (Hidalgo et al. 2004; Bell and Donoghue 2005; De Castro et al. 2025b) and representatives of other groups across Europe and America are missing. In this sense, our study represents a first step towards a better comprehension of plastome diversity in *Valeriana *sensu lato. About *V. trinervis*, the position in the phylogenetic trees is a preliminary but good representation of some distinctive features observed in respect to the plastomes of other *Valeriana* species.

### Repeat elements

Repeat sequences play an important role in generating variation in plastid genomes through point mutations, non-homologous recombination, and DNA polymerase slippage during replication (Ellegren 2002; George et al. 2015). Therefore, they are a valuable and highly utilised source of molecular markers for phylogeography and population genetics studies (e.g. Bucci et al. 2007; Semerikova et al. 2021). In our plastome comparisons, we found a marked a difference in the type and abundance of repeat elements. The plastome of *V. trinervis* contains the highest number of forward repeats (86.4%) and the lowest of palindromic repeats (13.3%). On the contrary, in the other *Valeriana* spp. forward repeats ranged from 56.6% to 76.4%, and palindromic repeats from 21.4% to 42.4% and conspecific samples were not comparable in these terms in accordance with the position in the phylogenetic trees. Regarding short tandem repeats, the difference between the analysed plastomes were less marked. The number of total SSRs (39) was in line with *Valeriana* taxa (36–39) except *V. officinalis* voucher B267 that presented the lowest number of SSRs (30) and a unique tri-nucleotide motif (AAT/ATT); instead, *V. trinervis* exhibited a unique AC/GT repeat motif. Similarly, long tandem repeats were comparable between *V. trinervis* and the other *Valeriana* spp. in the size range between 12 and 36 bp, but differences occurred for smaller and larger sizes. *Valeriana officinalis* B267 had unique LTRs (e.g. 42–46 and 52–56 bp) and, generally, larger sizes of repeats not observed in any other taxon. Compared to other Caprifoliaceae (e.g. *Lonicera* L. spp., *Weigela florida* (Bunge) A.DC.), which presented repeat units mainly in the range of 21–50 bp (Fan et al. 2018), we observed smaller LTRs (7–36 bp) in our target species. However, there is no common trend when comparing the number of repeats among congeneric species across plant families. For instance, in *Primula* L. (Primulaceae; Ren et al. 2018) and in *Ligustrum* L. (Oleaceae; Long et al. 2023), high differences in the number of long repeat sequences have been observed, while these differences were neglectable in *Bupleurum* L. (Apiaceae; Huang et al. 2021) and in *Wikstroemia* Spreng (Thymelaeaceae; Zhang et al. 2025).

### Codon usage and non-canonical start codons

Codon usage bias, i.e. the non-random use of synonymous codons within a coding sequence, is a universal feature of genomes originating from a balance between mutational patterns and natural selection (Athey et al. 2017). It plays an important role in a multitude of transcriptional and translational processes by affecting chromatin structure and mRNA folding (Quax et al. 2015; Liu 2020). Codon bias varies not only within and among species, genera and families, but also between genes and sites within a gene into a specific organism (Parvathy et al. 2022). In our study, 29 codons showed RSCU > 1, indicating a preference in their usage in respect to synonymous codons. This number is line with values (29–32 biased codons) found in other studies considering taxa within the same genus (Chen et al. 2024; Liu et al. 2024) or family (Shi et al. 2023; Li et al. 2024a, b). In addition, the codon preferences of *V. trinervis* and other *Valeriana* spp. were quite similar, suggesting a close relationship between them, as observed in some congeneric taxa (Jiang et al. 2023; Sudmoon et al. 2024).

Some plastid genes also presented non-canonical start codons, a feature observed in several groups from algae to angiosperms (Parvathy et al. 2022; Sadhu et al. 2023). The *ndh*D gene represents an interesting and well-studied case of non-canonical translation initiation, where an ACG codon replaces the canonical one AUG in many species (Martín and Sabater 2010). To carry out the translation processes, in some species *ndh*D transcripts undergo RNA editing to convert ACG to the canonical AUG start codon, while in others the translation starts from the ACG codon itself. For instance, in *Nicotiana tabacum* L. and *N. sylvestris* Speg. partial editing occurs, while in *N. tomentosiformis* Goodsp. the ACG codon remains completely unedited without hampering the normal translation (Zandueta‐Criado and Bock 2004). We found an ACG starting codon in the *ndh*D gene of *V. trinervis* but in none of the other *Valeriana* plastomes available in NCBI; however, we attribute this result to a likely mis-annotation rather than a real absence of non-canonical starting codons. Indeed, after re-annotating the deposited *Valeriana* plastomes with CPGAVAS2, we found non-canonical start codons in the *ndh*D genes of all taxa (see Supp. Information S3 – Suppl. Datasets S2–S8). Therefore, we recommend comparing different annotation tools before making conclusions. We detected also differences in the length of the two copies of *ndh*D in *V. trinervis* and all the other *Valeriana* taxa with two copies (all except *V. officinalis* B267 and SN0759). All the annotations of *ndh*D obtained with CPGAVAS2 were consistent with the gene boundaries found in other species and checked with BLAST searches. In addition, the translation of the nucleotide sequences before and after the detected boundaries contained stop codons or were not recognised as *ndh*D in BLAST searches. Therefore, we are confident that the annotations are correct and this discrepancy could be the result of ongoing pseudogenisation, but further evidence is needed to confirm or not this hypothesis. In addition to *ndh*D, other genes showing non-canonical start codons were *psb*C, *psb*L, and *rps*19. These findings were corroborated by some literature studies (e.g. Kuroda et al. 2007; Chen et al. 2018; Su et al. 2018; Zheng et al. 2019; Liew et al. 2022), but also reported for other genes too (e.g. Wang et al. 2020a, b; Pei et al. 2022). In general, the preferred start codon was ACG over ATG. The only exceptions were GTG in the *ndh*D gene of *V. officinalis* voucher SN0759, in the *psb*C gene of *V. officinalis* accession PQ030840 and in all *Valeriana* plastomes except *V. jatamansi* and *V. trinervis* for *rps*19, and TTG in the *psb*L of *V. officinalis* voucher SN0759. The reasons behind this non-canonical ATG start for some plastid genes is not fully understood yet, but it is known that the ACG codon can be converted into an AUG start codon through RNA editing (Neckermann et al. 1994). In addition, it has been reported that both ACG and GTG triplets can initiate translation with a strength varying from 15 to 30% of AUG activity, and the relative efficiency of ACG is lower than GUG (Mehdi et al. 1990; Su et al. 2018). We also found a non-canonical stop codon (CTA) for the second copy of *rps*12. As for *ndh*D, this is not explainable with mis-annotation because the translation of nucleotides after this point is not recognised as *rps*12 by BLAST searches and comparisons with other *Valeriana* sequences. Therefore, this could be a case of pseudogenisation too. To the best of our knowledge, no cases of AGT as stop codon have been reported in other plants.

### Hotspots of variation

One of the reasons behind the assembly of plastid genomes and their comparisons with other species is to find variable regions to be used for phylogenetic and phylogeographic studies. Due to the stable genetic structure, high copy number, absence of (or very rare) recombination, and uniparentally transmission, the plastid genome has been and still is the preferred target for evolutionary studies in plants (Dobrogojski et al. 2020). Several fragments of coding regions, introns, and intergenic spacers, like *atpB-rbcL*, *matK*, *rbcL*, *rps16*, *trnH-psbA*, *trn*Q-*rps*16, *trnL-F*, *trnS-G*, etc., have been used in phylogenetic and phylogeographic studies (e.g. De Castro et al. 2012, 2020; De Luca et al. 2022, 2024), and some of them (e.g. *matK*, *rbcL*, *trnH-psbA*) have been proposed as markers for plant DNA barcoding (CBOL Plant Working Group 2009; Hollingsworth et al. 2016; De Castro et al. 2017). Through the alignment of *V. trinervis* and the other *Valeriana* plastomes (Fig. 2), we identified several potential markers for phylogenetic studies: the genes *acc*D, *pet*D, *rpo*C1, *rps*18, and *ycf*1; the intergenic spacers *acc*D-*rbc*L, *ndh*I-*ndh*G, *psb*J-*pet*A, and *trn*V-UAC-*ndh*C; and *ycf*1-*rps*15 and the *clp*P introns, some of which have already been implemented in De Castro et al. 2025a, b (a, b) for *Centranthus* phylogeny and the phylogeography of *Centranthus* taxa belonging to sect. *Nervosae*. Variation in these regions was both in terms of single nucleotide polymorphisms and indels and could be particularly useful for future studies in this complex family.

## Conclusions

In this study, we assembled the complete plastid genome sequence of the critically endangered *V. trinervis* by Illumina sequencing of WGS data. After annotating and reporting its features, we compared it with the plastome of other *Valeriana* species and accessions to identify differences and similarities in gene content, repeat regions, and codon usage. Regarding repeated regions, the plastome of *V. trinervis* contained the highest proportion of forward repeats and the lowest of palindromic repeats compared to *Valeriana* plastomes. On the contrary, the number of microsatellite repeats in *V. trinervis* was similar to all the other *Valeriana* taxa except *V. officinalis* voucher B267. Codon usage was comparable across all plastid coding genes. Some non-canonical start codons were observed and not shared by all taxa. From the phylogenetic point of view, the plastid sequence of *V. trinervis* was sister to all Eurasian *Valeriana* spp. In addition, several markers presented high levels of variation that make them useful candidates for future phylogenetic studies.

## Supplementary Information

Below is the link to the electronic supplementary material.

Supplementary file1 (PDF 1341 KB)

Supplementary file2 (XLSX 58 KB)

Supplementary file3 (XLSX 1220 KB)

## Data Availability

The complete plastid genome of *V. trinervis* is available in NCBI GenBank under the accession number PV026107. All the data not published in the text are available as supplementary material on the publisher’s website.

## Abbreviations

IR: Inverted Repeat
LSC: Large Single Copy
RSCU: Relative Synonymous Codon Usage
SSC: Small Single Copy
SSR: Simple Sequence Repeat
