# Supplementary material for: The plastid genome of the critically endangered Valeriana trinervis (= Centranthus trinervis) and insights from comparison with other Valeriana plastomes (Caprifoliaceae)
Source: Planta. 2025 Sep 10;262(4):100. doi: 10.1007/s00425-025-04815-w (PMC12423159; doi:10.1007/s00425-025-04815-w)

Supporting Information S1 for:

The plastid genome of the critically endangered *Valeriana trinervis* (= *Centranthus trinervis*) and insights from comparison with other *Valeriana* plastomes (Caprifoliaceae).

Planta

Daniele De Luca[1]*, Olga De Castro[2, 3]*

[1]Department of Humanities, University of Naples Suor Orsola Benincasa, Via Santa Caterina da Siena 37, I-80132, Naples, Italy (daniele.deluca@unisob.na.it; daniele.deluca088@gmail.com)

[2]Department of Biology, University of Naples Federico II, Via Cinthia 26, I-80126, Naples, Italy (odecastr@unina.it; odecastr@gmail.com)

[3]Botanical Garden of Naples, University of Naples Federico II, Via Foria 223, I-80139 Naples, Italy

(* = corresponding author)

Supplementary Figure S1. Coverage plot illustrating the sequencing depth of the raw reads mapped against the assembled plastome of *Valeriana trinervis*.

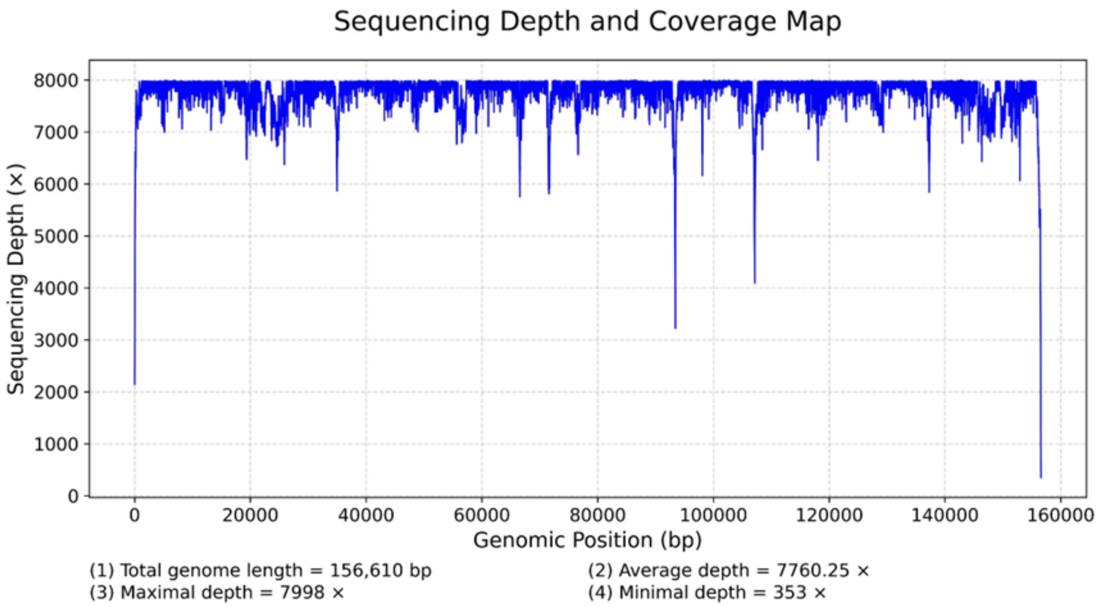

Supplementary Figure S2. Comparison of the plastome of *Valeriana trinervis* (used as reference) against the ones of the other *Valeriana* spp. using mVISTA. The y-axis reports, for each row, the percent of identity in the 50-100% range. CNS stands for conserved non-coding sequence.

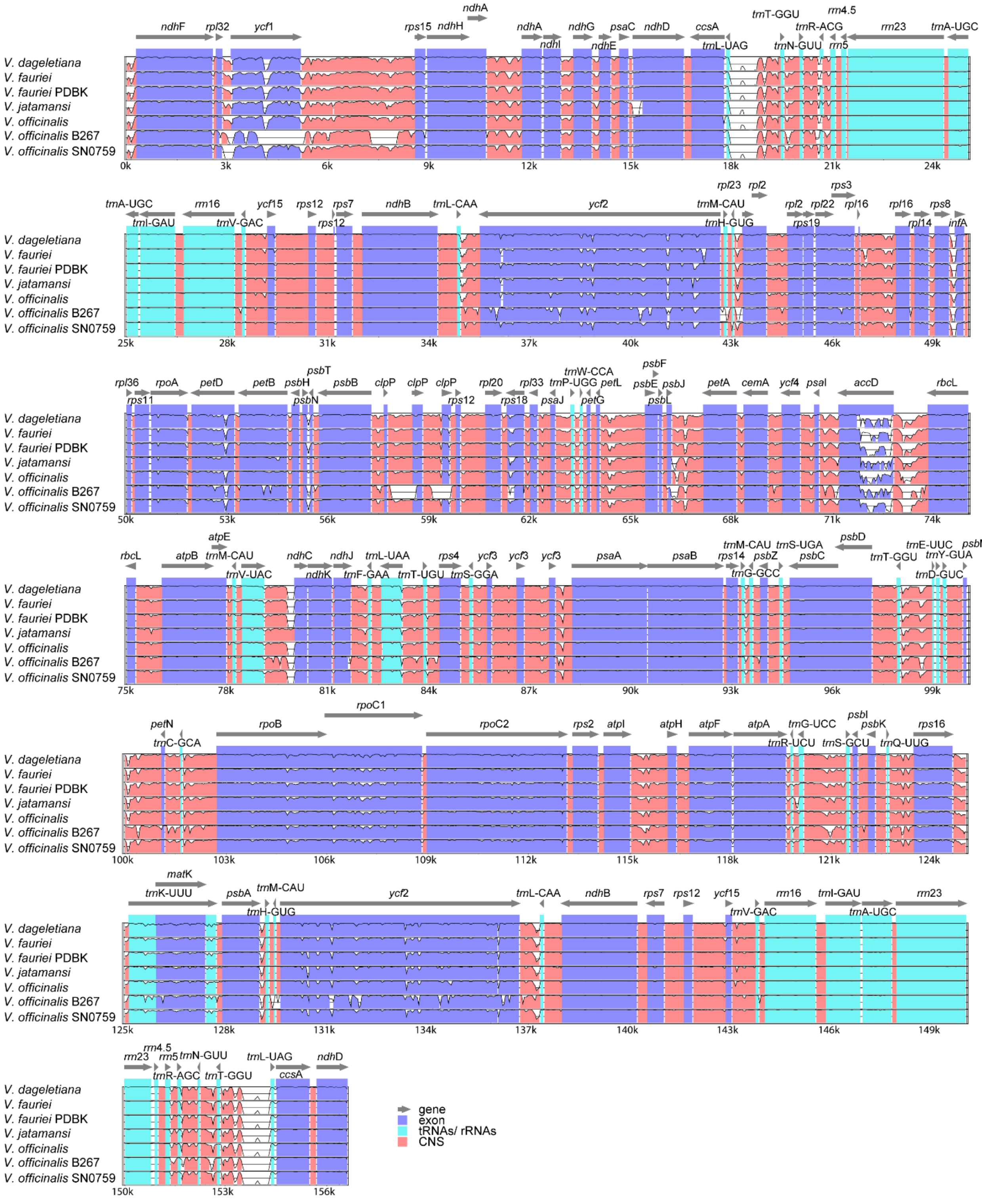

Supplementary Figure S3. Junction sites in the plastomes of *Valeriana trinervis* and other *Valeriana* spp. JLB = junction between LSC and IRb; JSB = junction between SSC and IRb; JSA = junction between SSC and IRa; JLA = junction between LSC and IRa.

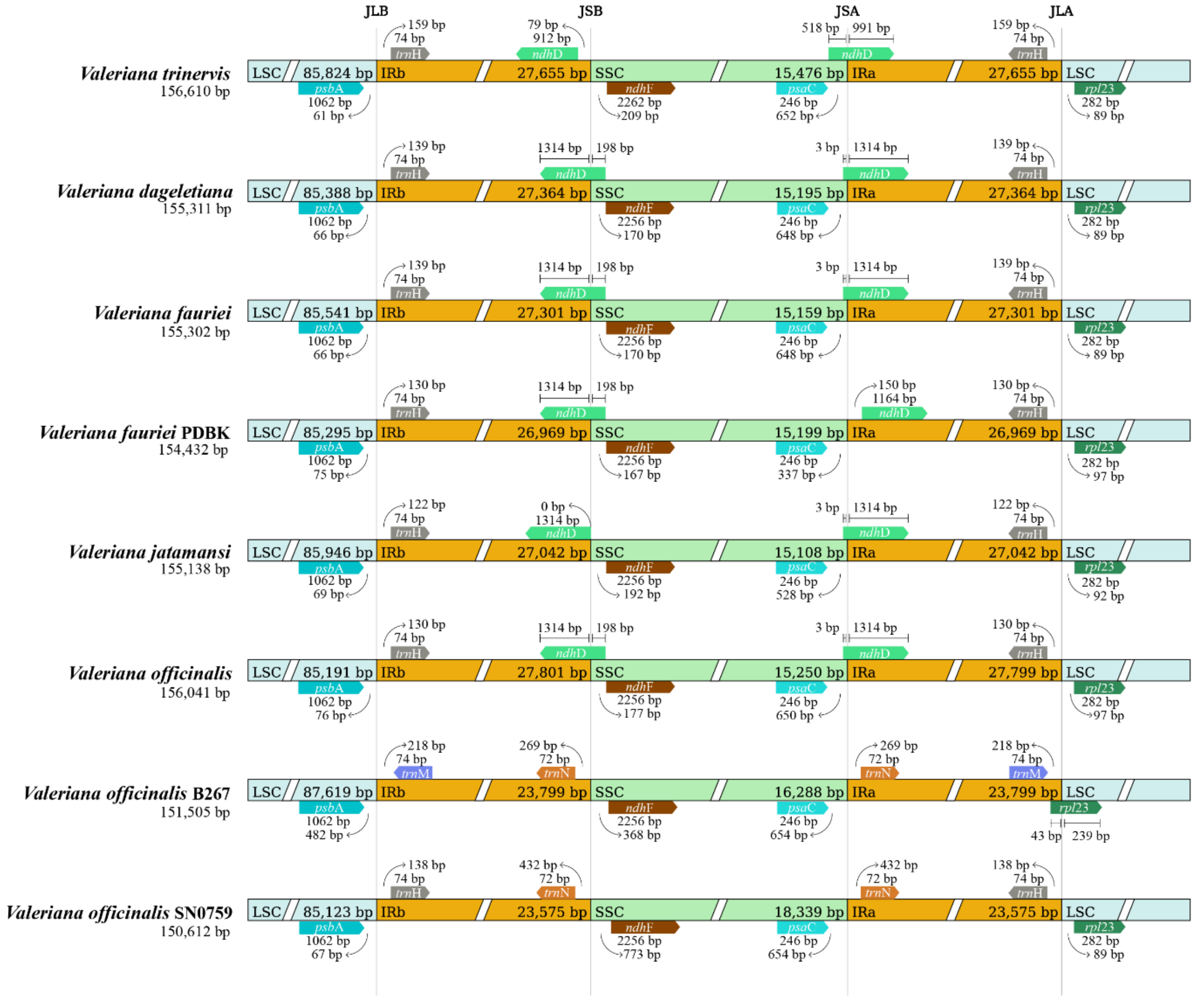

Supplementary Figure S4. Heatmap of codon usage in protein-coding genes in the plastomes *of Valeriana trinervis* and other *Valeriana* species.

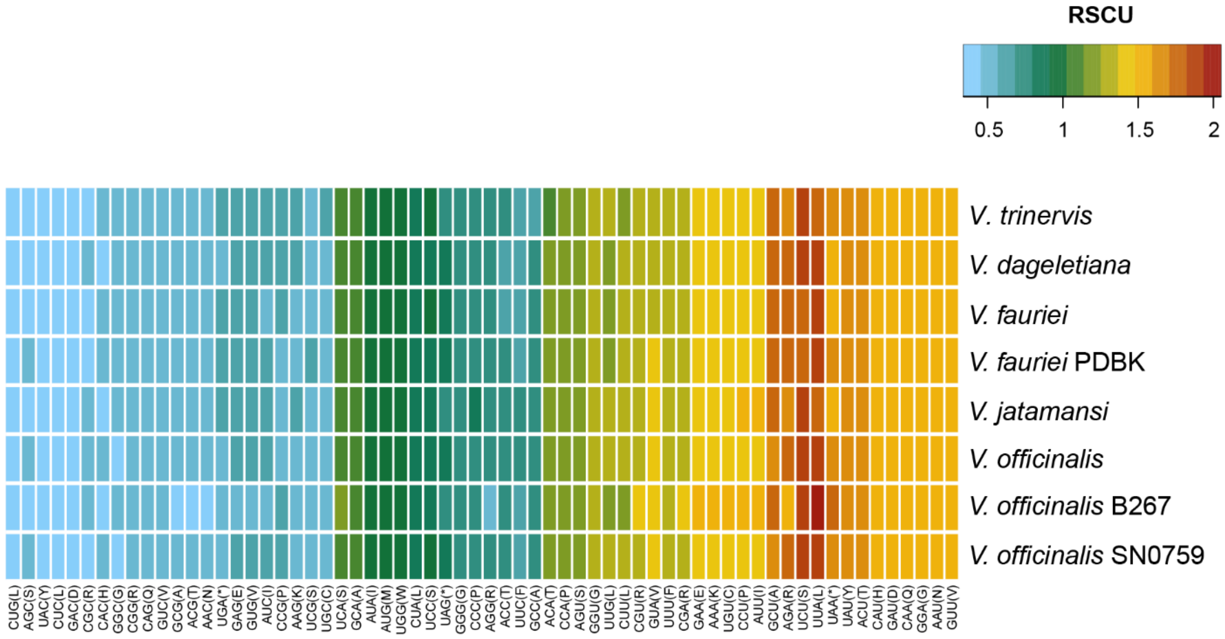

Supplement: Supplementary file 1 — Supplementary file1 (PDF 1341 KB) [file 425_2025_4815_MOESM1_ESM.pdf]
